# Do small changes in rotation affect measurements of lower extremity limb alignment?

**DOI:** 10.1186/s13018-017-0571-6

**Published:** 2017-05-22

**Authors:** Amir A. Jamali, John P. Meehan, Nathan M. Moroski, Matthew J. Anderson, Ramit Lamba, Carol Parise

**Affiliations:** 1 Joint Preservation Institute, 2825 J Street, Suite 440, Sacramento, CA 95816 USA; 2 UC Davis Medical Center, 4860 Y St., #4800, Sacramento, CA 95817 USA; 3 Department of Orthopaedic Surgery, University of California, Irvine, 101 The City Drive South, Pavillion III, Building 29A, Orange, CA 92868 USA; 4 UC Davis Department of Orthopaedics, 4635 2nd Ave, Research 1 Room 2000, Sacramento, CA 95817 USA; 5 UC Davis Department of Radiology, 4860 Y St., #3100, Sacramento, CA 95817 USA; 6 Sutter Institute for Medical Research, 2801 Capitol Ave Suite 400, Sacramento, 95816 USA

**Keywords:** Alignment, Lower extremity, Osteotomy, Rotation, Constitutional varus, Total knee replacement

## Abstract

**Background:**

The alignment of the lower extremity has important implications in the development of knee arthritis. The effect of incremental rotations of the limb on common parameters of alignment has not been studied. The purpose of the study was to (1) determine the standardized neutral position measurements of alignment and (2) determine the effect of rotation on commonly used measurements of alignment.

**Methods:**

Eighty-seven full length CT angiography studies (49 males and 38 females, average age 66 years old) were included. Three-dimensional models were created using a rendering software program and placed on a virtual plane. An image of the extremity was obtained. Thirty scans were randomly selected, and those models were rotated in 3° intervals around the longitudinal axis and additional images were obtained.

**Results:**

In the neutral position, the mechanical lateral distal femoral articular angle (mLDFA) was 85.6 ± 2.3°, medial proximal tibial angle (MPTA) was 86.1 ± 2.8°, and mechanical tibiofemoral angle (mTFA) was −0.7 ± 3.1°. Females had a more valgus alignment with a mTFA of 0.5 ± 2.9° while males had a more varus alignment with a mTFA of −1.7 ± 2.9°. The anatomic tibiofemoral angle (aTFA) was 4.8 ± 2.6°, the anatomic lateral distal femoral angle (aLDFA) measured 80.2 ± 2.2°, and the anatomical-mechanical angle (AMA) was 5.4 ± 0.7°. The prevalence of constitutional varus was 18%.

The effect of rotation on the rotated scans led to statistically significant differences relative to the 0° measurement for all measurements. These effects may be small, and their clinical importance is unknown.

**Conclusions:**

This study provides new information on standardized measures of lower extremity alignment and the relationship between discreet axial rotations of the entire lower extremity and these parameters.

## Background

The alignment of the lower extremity has been an area of ongoing study for decades. Standard radiographs have been used to determine the “normal” parameters of alignment of the lower extremity. These are prone to technical errors based on distance from the cassette and rotation of the lower extremity around the longitudinal axis. Deviations from “normal” have been broadly categorized at malalignment although a clear definition of “normal alignment” has not been established. One can define “normal” on a statistical basis as lying within some arbitrarily defined range relative to the mean or on a pathological basis according to the risk of the joint undergoing degeneration secondary to the deformity. Malalignment of the native lower extremity has been associated in previous studies with a higher risk of osteoarthritis [1–4]. Accurate preoperative and postoperative alignment parameters are required for planning and prediction of outcome for both osteotomies and total knee replacement [5–11]. Thus, the assessments of both the lower extremity alignment in native knees and those that have undergone replacement depend on an accurate definition of native lower extremity alignment [12, 13].

In spite of increased sophistication in imaging and computer generated reconstructions, most surgeons still depend on two-dimensional radiographs in planning operations such as osteotomy, unicompartmental knee replacement, or total knee replacement.

The objectives of this study were twofold. We sought to (1) determine the standardized neutral position measurements of alignment and (2) determine the effect of rotation on commonly used measurements of alignment. We prepared three-dimensional models of the lower extremity in a standardized position and rotated the models in 3° increments in each direction, taking digital photographs in each position.

## Methods

A total of 221 full lower extremity CT angiography studies for vascular disease workup were performed at our institute between July 8, 2008, and May 14, 2010. Of these, 87 patients (49 males and 38 females) were included in the present study. The average age was 66 years old (range 28–91 years old) Exclusion criteria included advanced osteoarthritis of the hip, knee, or ankle, radiographic evidence of previous realignment surgery or fracture, irregular positioning in the scanner, or any type of lower extremity joint prosthesis.

### Normal values of coronal alignment of the lower extremities with the femur placed on a virtual flat table

The first portion of the study was the determination of the normal values of coronal alignment of the lower extremity without the effect of rotation and in a neutral position. Three-dimensional models were created from the CT data using a commercially available and previously validated three-dimensional rendering software program (Mimics, Materialise, Ann Arbor, MI) [14]. These models were then placed on a virtual flat table in the computer environment. The femora rested with the virtual table plane passing through the posterior most point of the greater trochanter and the posterior most points of both the medial and lateral femoral condyles. In this neutral position, a high resolution image of the femur and the tibia from anterior to posterior was obtained. Next, 30 scans were randomly selected, and using the software, the entire lower extremity model was rotated in discreet 3° intervals in both internal and external rotation around the virtual axis from the femoral head to the center of distal femur up to 12° in each direction. After each rotation, a new anterior to posterior image of the now rotated lower extremity was obtained. The image files were then analyzed using a custom measurement analysis program written in Matlab (Mathworks, Natick, MA, USA). The analysis was performed in this fashion to optimize speed and precision and minimize risk of observer bias. The independent variable was the degree of rotation. The dependent variables were the alignment parameters measured.

### Definitions for image analysis, points, axes, and angular measurements

#### Points

The convention used by Moreland et al. was utilized to define the center of the femoral trochlea as the center point of the knee [15]. Moreland et al. had described taking a visual midpoint among a total of five points to define the center of the knee. These included the center of the femoral notch (trochlea), center of tibial spines, center of femoral condyles, center of soft tissue, and center of the tibia. They found that all points were within 5 mm of one another. Based on the high consistency of the center of the femoral trochlea, we chose that point as the center of the knee. The center of the ankle was defined visually as the center of the distal tibial articular surface. The other points selected on lower extremity images obtained were the center of the femoral head, the most distal points of the distal medial and lateral femoral condyles, and the most proximal point of the medial and lateral tibial plateaus.

The proximal femoral shaft center (PFSC) was defined by selecting two lateral points and two medial points in the subtrochanteric region of the femur and allowing the software to calculate the geometric center of those four points located centrally within the femoral shaft in the subtrochanteric region.

### Axes

#### Mechanical axes

The mechanical axis of the femur was defined as a line drawn from the center of the femoral head to the center of the knee. The mechanical and anatomical axes of the tibia were both defined in an identical fashion as the line connecting the center of the knee and the center of the ankle.

#### Anatomical axes

The line connecting the PFSC and the center of the femoral trochlea was used to define the anatomical axis of the femur. The line connecting the center of the femoral trochlea and the center of the ankle was used to define the anatomical and mechanical axes of the tibia as noted above.

#### Articular axes

The distal femoral articular axis was defined by the line connecting the distal most points of the medial and lateral femoral condyles. The proximal tibial articular axis was defined as the line connecting the two most proximal points of the tibial plateaus.

### Angular measurements

#### Mechanical angle measurements

**Fig. 1:**
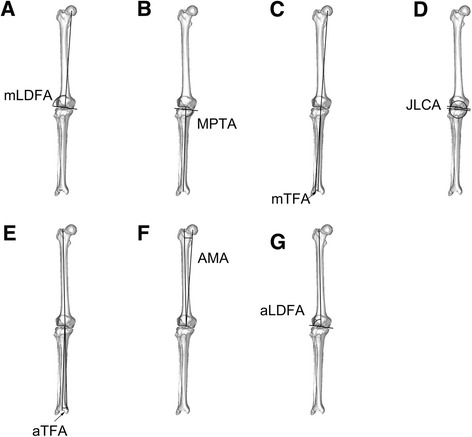
Measurement methods of common parameters of lower extremity alignment studied in this paper. **a** mLDFA. **b** MPTA. **c** mTFA. **d** JLCA. **e** aTFA. **f** AMA. **g** aLDFA

The mechanical lateral distal femoral articular angle (mLDFA) was defined as the lateral angle between the femoral mechanical axis and the distal femoral articular axis [16] (Fig. 1a). The medial proximal tibial angle (MPTA) was unique among the measurements in that it was included in both the mechanical parameters and the anatomical parameters and was defined as the medial angle between the mechanical (as well as anatomical) axis of the tibia and the proximal tibial articular axis (Fig. 1b). The mechanical tibiofemoral angle (mTFA) [16] was defined as the angle between the femoral mechanical axis and the tibial mechanical axis with a positive value indicative of a valgus alignment and a negative value indicative of a varus alignment of the lower extremity (Fig. 1c). The joint line convergence angle (JLCA) was defined as the angle between the proximal tibial and distal femoral articular axes with a negative value indicative of convergence laterally and a positive value indicative of convergence medially (Fig. 1d).

#### Anatomical angle measurements

The anatomic tibiofemoral angle (aTFA) [16] was defined as the angle between the anatomical axis of the femur and the anatomical-mechanical axis of the tibia (Fig. 1e). Once again, a positive value was indicative of a valgus and a negative value indicative of a varus alignment of the lower extremity. The angle between the mechanical and anatomical axes of the femur was defined as the anatomical-mechanical angle (AMA) [16] (Fig. 1f). The anatomical lateral distal femoral angle (aLDFA) was defined as the angle between the anatomical axis of the femur and the distal femoral articular axis [16] (Fig. 1g).

The anatomical medial proximal tibial angle was by convention defined to be equivalent to the medial proximal tibial angle (MPTA) due to the equivalence of the mechanical and anatomical tibial axes as noted above.

#### Effect of 3° rotational intervals on coronal alignment

The rotated anterior to posterior (AP) images of the 3D models of 30 randomly selected specimens were analyzed in the same fashion as the neutral AP images described above. By convention, negative measurements indicated the lower extremity to be internally rotated and positive measurements externally rotated around the longitudinal axis of the femur.

### Statistical analysis

Inter- and intraobserver reliability analysis was performed for each parameter using intraclass correlation coefficients (ICC) for intraobserver reliability analysis for observer 1 (AAJ) at two time points 4 weeks apart and interobserver reliability between the two observers (AAJ and MA) for a total of 20 of subjects. All ICCs were greater than 0.94 indicating excellent reliability with the exception of the measurements of JLCA with ICC of 0.18 and 0.68 for inter- and intraobserver reliability respectively. The effect of rotation of each parameter was analyzed using repeated measures analysis of variance (RMANOVA) with Bonferroni correction as appropriate. The prevalence of constitutional varus based on gender was analyzed using the chi-squared test. All analyses were performed with SPSS (IBM, Chicago, IL), Excel (Microsoft, Redmond, WA), and StatView software (SAS, Cary, NC). Statistical significance was set at *p* < 0.05.

## Results

### Mechanical measurements

**Fig. 2:**
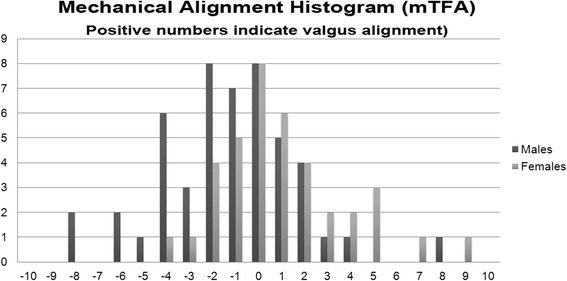
Histogram of mechanical tibiofemoral angle (mTFA) in patients in the series subcategorized by gender

The mLDFA was 85.6 ± 2.3°. The MPTA was 86.1 ± 2.8°. The JLCA angle was −1.2 ± 1.7°. The mechanical tibiofemoral angle (mTFA) was −0.7 ± 3.1°. Females had a more valgus alignment with a mTFA of 0.5 ± 2.9° while males had a more varus alignment with a mTFA of −1.7 ± 2.9° (Fig. 2) (Table 1).

**Table 1:** Summary of lower extremity alignment measurements from this study (n=87)

Mechanical alignment parameters	
Mechanical lateral distal femoral articular angle (mLDFA)	85.6 ± 2.3°
Medial proximal tibial angle (MPTA)	86.1 ± 2.8°
Mechanical tibiofemoral angle	−0.7 ± 3.1°
The joint line convergence angle (JLCA)	−1.2 ± 1.7°
Anatomical alignment parameters	
Anatomic tibiofemoral angle (aTFA)	4.8 ± 2.6°
Lateral distal femoral angle (aLDFA)	80.2 ± 2.2°
Medial proximal tibial angle (MPTA)	86.1 ± 2.8°
Other	
Anatomical-mechanical angle (AMA).	5.4 ± 0.7°

### Constitutional varus

The prevalence of constitutional varus defined as mTFA of more than 3° varus was 16/87, or 18%. There was a statistically significant difference (*p* = 0.005) in the prevalence based on gender with 2/38, or 5% of females, and 14/49, or 29% for males, being classified as constitutional varus.

### Anatomical measurements

The anatomic tibiofemoral angle (aTFA) was 4.8 ± 2.6°. The anatomic lateral distal femoral angle (aLDFA) measured 80.2 ± 2.2°. The medial proximal tibial angle (MPTA) was 86.1 ± 2.8°. The anatomical-mechanical angle (AMA) was 5.4 ± 0.7°.

### Effect of rotation

**Fig. 3:**
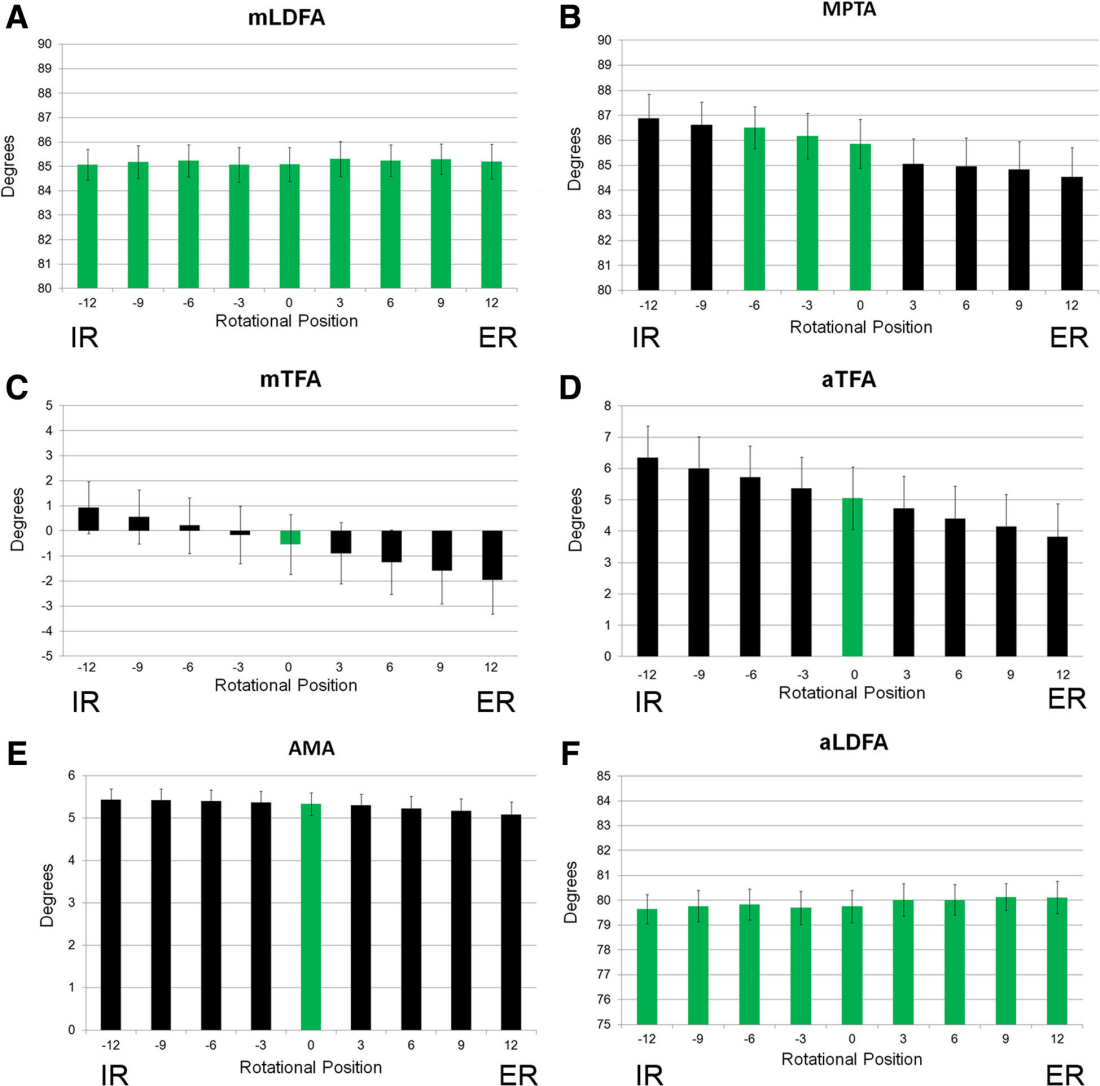
**a**–**f** Effect of rotation on various measured parameters of lower extremity alignment (*n* = 30). Degrees for each parameter ±S.D. (*Green* indicates no significant difference relative to baseline or 0° measurement)

The effect of rotation in 3° increments led to a statistically significant difference in measurements for all measurements except for aLDFA and mLDFA. For aLDFA and mLDFA, there was no significant difference in spite of rotation of the images. For the remaining parameters, the effect of rotation varied. Measurements taken with the lower extremity rotated as little as 3° leading to significant differences (Fig. 3). For the mTFA, aTFA, and AMA measurements, pairwise comparisons indicated that all rotated measurements were significantly different from the neutral, 0°, rotational position. This indicated that even a 3° rotational variation in these parameters leads to a statistically significant difference in the measured value. For MPTA, measurements at the −3° and −6° positions were not significantly different than those at the neutral position while all others were significantly different than the neutral position value. JLCA was not analyzed due to the low interobserver reliability of that measurement.

## Discussion

The objective of this study was to twofold. The first goal was to obtain commonly performed measurements of alignment using standardized three-dimensional lower extremity models in a neutral position and to compare those values to data previously reported in the literature. Based on our analysis of the available literature, the measurements obtained in the study were comparable to those of the previous literature (Table 2) [1, 15, 17–20]. Our second goal was to determine the effect of rotation of the lower extremity, even in as little as 3° intervals, on these commonly performed measurements of alignment. We found that small amounts of rotation did lead to statistically significant differences for a number of parameters analyzed including mTFA, aTFA, and AMA and to a limited degree for MPTA.

**Table 2:** Literature review

Reference	Number of normal subjects	Mean age	Gender	Technique	aLDFA	mLDFA	MPTA	AMA	mTFA	JLCA
Present study	87 patients undergoing CT angiography	N/A	49M, 38F	3D CT reconstructions with computer assisted analysis	80.24 ± 2.16°	85.59 ± 2.26°	86.09 ± 2.77°	5.35 ± 0.68°	−0.71 ± 3.07°	−1.21 ± 1.73°
3	119 healthy volunteers	38	52M, 67F	Standard full length radiographs	N/A	86.04–2.33°	86.92 ± 2.33°	N/A	−0.97 ± 2.86°	−1.85 ± 1.61°
15	25 male volunteers	30	25M, 0F	Standard full length radiographs	N/A	N/A	87.2°	4.05°	−1.3°(varus)	N/A
18	50 healthy Chinese adult volunteers	23-year-old females, 24-year-old males	25M, 25F	Standard full length radiographs	N/A	N/A	85.1 (males), 84.6 (females)	3.6 males, 3.1 females	−2.2 (males and females)	N/A
17	120 healthy Caucasian adult volunteers	range 25–60	60M, 60F	Standard full length radiographs	N/A	88.13°	86.85°	4.9 ± 0.7°	−1.2 ± 2.2°	N/A
20	100 healthy Iranian adult volunteers	range 15–32	50M, 50F	Standard full length radiographs	83.2 ± 3°	N/A	87.2	5.7 ± 1.2°	−1.5°(varus)	−1 ± 1.6°
21	118 healthy female Korean volunteers	range 20–39	118F	Standard full length radiographs	N/A	87.78 ± 1.68°	86.82 ± 1.61°	5.99 ± 0.7°	−1.35 ± 2.04°	N/A

Bellemans et al. performed a study using standard radiographs positioned according to the methodology of Paley [16]. The study consisted of 250 volunteers, 125 male and 125 female. They measured many of the same parameters as in our study. Their main finding of their study was that 32% of males and 17% of females fit the criteria of “constitutional varus” defined as a hip-knee-ankle (HKA, equivalent to mTFA in this study) angle of 3° or greater varus. They further postulated that correcting such patients to a neutral mechanical axis through total knee replacement may lead to unfavorable outcomes. The authors noted one of the weaknesses of their study was the use of plain radiographs. They indicated that the rotational position of the extremities was controlled by positioning the subjects standing barefoot with the feet together and “standing at attention” with the patella oriented forward. Although this is a widely used technique, it is clear that all studies that use this methodology are at best estimating at the degree of rotation of the extremities. They considered the use of CT scans but were concerned about increased radiation. Our study addressed the issues discussed by Bellemans et al. By using CT scan generated models using preexisting scans, we avoided any additional radiation to patients. We also addressed the rotational variability of the positioning by placing the models on virtual planes and taking images in this position for the semiautomatic analysis. Song et al. explored the incident of constitutional varus in a controlled population of normal volunteers demonstrating a 20% incidence of constitutional varus in a group of Korean female volunteers [21]. Our data demonstrated a lower incidence of constitutional varus than both of these studies. This may reflect the different methodologies used (CT generated 3D models vs standard radiographs) as well as a different population (Belgian and Korean populations compared to a population from the USA).

A number of studies have evaluated the effect of rotation on lower extremity alignment parameters. Oswald et al. performed a cadaveric study on 38 lower extremity specimens using standard radiographs and analyzed the effect of rotation on the anatomic mechanical angle (AMA) [22]. At neutral, the AMA was 6.3°. They noted a change in this angle from 6.8° in 15° of internal rotation to 5.7° in 15° of external rotation for an average decrease in the AMA of 0.036° per degree of rotation. Radtke et al. performed a limited study on one lower extremity saw bone model in which a total knee replacement was implanted [23]. They then took five series of X-rays of that specimen measuring the AMA in various rotations from 20° of internal rotation (6.83°) to 20° of external rotation (4.63°) for an average decrease of 0.055° per degree of rotation. In our series, the AMA in the rotated studies was 5.33° at neutral and decreased from 5.43° to 5.08° between 12° of internal rotation and 12° of external rotation for an average decrease of 0.0146° per degree of rotation.

This study has a number of important weaknesses. The population included in this study was a random sample of patients undergoing CT angiography for vascular disease. As a result, the majority of the subjects were elderly with an average age of 66 years old. Furthermore, the characteristics of the population based on factors such as weight, height, or race could not be determined. In spite of our efforts to rule out patients with osteoarthritis of the knee, undoubtedly some degree of degeneration may have been present in this population. Second, we standardized the position of the femur relative to the coronal plane. However, there is some variability of the position of the tibia relative to that of the femur. We chose to standardize the femur since the methods for doing so are more reproducible based on the three points of contact of the femur on the coronal plane, namely the medial and lateral posterior condyles and the posterior greater trochanter. We operated under the assumption that the tibia would be relatively consistent in relation to the femur. Third, the software output was a three-dimensional reconstruction rather than a radiograph. These images do not demonstrate typical physical distortions such as parallax seen in standard radiographs, potentially limiting their comparisons to the traditional methods of alignment measurement used in previous publications. Fourth, in spite of our use of large monitor computer workstations, there is a possibility of some degree of inaccuracy of the measurements of the most distal aspect of each condyle and most proximal aspect of each tibial plateau due to the relatively low magnification of these regions relative to the entire image of the lower extremity. The number of patients included in the study was relatively low compared to some other population based studies. Another limitation of this study is that although there is a statistically significant error introduced with rotation of the leg for some of the parameters such as MPTA, the size of this error may not be clinically significant. It rests upon the reader to determine for themselves the clinically relevant range of error acceptable to them from a clinical perspective for the analysis or operation being considered. Some procedures may be more sensitive to variabilities in alignment than others.

In spite of these weaknesses, this study is one of the first to provide rotationally controlled values for commonly performed measurements of coronal alignment and one of the few studies that indicates the sensitivity of commonly performed measurements of coronal alignment to discreet small rotations of the entire lower extremity.

## Conclusions

In conclusion, the current study validates the lower extremity alignment measurements that are commonly used in the fields of rheumatology and orthopedics with a more robust methodology that standardizes the rotational position of the lower extremity using three-dimensional models in a virtual environment. The study indicates that for some parameters, even a 3° rotational deviation can lead to a statistically significantly different value.

## Abbreviations

aLDFA: Anatomical lateral distal femoral articular angle. The angle between the anatomical axis of the femur and the distal femoral articular axis
AMA: Anatomical-mechanical angle. The angle between the mechanical and anatomical axes of the femur
aTFA: Anatomic tibiofemoral angle. The angle between the anatomical axis of the femur and the mechanical/anatomical axis of the tibia
CT: Computerized tomography
HKA: Hip-knee-ankle angle. Equivalent to mTFA in this study
ICC: Intraclass correlation coefficients
JLCA: Joint line convergence angle. The angle between the proximal tibial and distal femoral articular axes
mLDFA: Mechanical lateral distal femoral articular angle. The lateral angle between the mechanical axis of the femur and the distal femoral articular axis
MPTA: Medial proximal tibial angle. The medial angle between the mechanical/anatomical axis of the tibia and the proximal tibial articular axis
mTFA: Mechanical tibiofemoral angle. The angle between the mechanical axis of the femur and the mechanical/anatomical axis of the tibia
PFSC: Proximal femoral shaft center. The geometric center of four points, two points on the lateral femoral surface and two points on the medial femoral surface in the subtrochanteric region of the femur
RMANOVA: Repeated measures analysis of variance
